# Prevalence and determinants of HIV among reproductive-age women (15–49 years) in Africa from 2010 to 2019: a multilevel analysis of demographic and health survey data

**DOI:** 10.3389/fpubh.2024.1376235

**Published:** 2025-01-24

**Authors:** Alemu Gedefie, Amare Muche, Anissa Mohammed, Aznamariam Ayres, Dagnachew Melak, Eyob Tilahun Abeje, Fekade Demeke Bayou, Fekadeselassie Belege Getaneh, Lakew Asmare, Abel Endawkie

**Affiliations:** ^1^Department of Medical Laboratory Science, College of Medicine and Health Sciences, Wollo University, Dessie, Ethiopia; ^2^Department of Epidemiology and Biostatistics, School of Public Health, College of Medicine and Health Sciences, Wollo University, Dessie, Ethiopia; ^3^Department of Pediatrics and Child Health Nursing, College of Medicine and Health Sciences, Wollo University, Dessie, Ethiopia

**Keywords:** Africa, DHS, HIV, multilevel analysis, reproductive-age women

## Abstract

**Background:**

Human immunodeficiency virus (HIV) remains the leading cause of global morbidity and mortality. The incidence of HIV is disproportionately higher in Sub-Saharan regions, particularly the Southern African sub-region, which is the most affected region and accounts for 77% of all new HIV infections in the region. Thus, the aim of this study was to identify the determinants of HIV among reproductive-age women in Africa.

**Methods:**

This study was conducted among reproductive-age women in Africa, based on secondary data obtained from the Demographic Health Survey (DHS) conducted between 2010 and 2019. The outcome variable was HIV status, while individual- and community-level variables served as potential predictors. The model fit was assessed using Akaike’s Information Criterion, Bayesian Information Criterion, and − 2 Log likelihood. Then, multilevel mixed-effects analysis was used. Intra-cluster correlation coefficient, median odds ratio, and proportional change in variance were used to measure heterogeneity between clusters.

**Results:**

A total of 292,810 unweighted and 293,773 weighted reproductive-age women in 26 African nations were included in this study. The overall prevalence of HIV among reproductive-age women in Africa was 4.34% (95% CI: 4.2, 4.4%). The highest percentage of HIV was found in Lesotho (23.98%), followed by South Africa (19.12%), and Mozambique (14.67%). However, the lowest HIV prevalence was found in Niger (0.54%), Senegal (0.59%), and Burundi (0.79%). Southern Africa has the highest HIV burden (18.5%), followed by Eastern Africa (6.1%), while Western African countries have the lowest HIV burden. Increasing maternal age, higher maternal education, women who were unemployed, a history of multiple sexual partners, women in a union, community-level educational status, community-level wealth index, African sub-region, and urban residence were found to be independent predictors of HIV infection in Africa.

**Conclusion:**

The burden of HIV has remained higher, highlighting the need for targeted public health intervention strategies to prevent the transmission of HIV among key populations.

## 1 Introduction

Human immunodeficiency virus (HIV) is a type of virus that attacks the human immune system, causing a decrease in CD4+ cell counts and immune function, which leads to AIDS and other life-threatening opportunistic infections (1). Globally, since the start of the HIV epidemic, approximately 85.6 million individuals have been infected with HIV, and 40.4 million have died from AIDS-related illnesses. According to recent evidence, an estimated 39 million people were living with HIV (PLWHIV) in 2022, of whom approximately 1.3 million were newly infected. Global HIV and AIDS statistics show that women and girls accounted for 53% of all PLWHIV and 46% of all new infections in 2022. Surprisingly, young women and adolescent girls accounted for more than 77% of all new HIV infections in Sub-Saharan Africa (SSA) (2). Moreover, out of the global new HIV infections that were reported per day, two out of three cases were reported in SSA (3), and the Joint United Nations Program on HIV/AIDS (UNAIDS) reports that three new HIV infections and one AIDS-related death occur every minute (4). The likelihood of acquiring HIV in these target groups was more than three times higher than among their male peers (2).

Across different geographies, national boundaries, and even within individual provinces, the HIV epidemic has shown striking variation (5). The largest concentration of HIV-positive individuals is found in SSA, especially in Southern Africa (6). However, considering population size, the Northeast has the highest prevalence of HIV-positive individuals. Research from Southern Africa indicates that sexual interactions between adolescent girls or young women and older men are a common way of HIV transmission. Furthermore, children born to HIV-positive mothers are at risk of contracting the virus if their mothers are not receiving effective treatment or are not being monitored (6, 7).

Globally, HIV continues to be the primary cause of morbidity and mortality. There is mounting evidence that HIV/AIDS-related maternal deaths have significantly increased; however, it is challenging to determine the true contribution of HIV/AIDS to maternal mortality because pregnant women’s HIV status is not usually recognized. This causes an underestimation of the data. AIDS has surpassed direct obstetric reasons as the primary cause of maternal mortality in several countries with high HIV incidence (8, 9). Different factors have been associated with the escalating prevalence of HIV among reproductive-age women. Some of the drivers of HIV are women who are separated from their spouses, and who travel long distances to reach health facilities, with a low level of wealth, and poor media exposure (9).

As a long-time partner of the Global Fund to Fight AIDS, the United Nations Development Program (UNDP) remains committed to helping nations achieve the Sustainable Development Goals (SDGs) through its HIV, Health Strategy, and Strategic Plan 2022–2025 (10). However, inequalities hamper the AIDS response, with key populations accounting for 70% of new HIV infections globally and 51% of new infections in sub-Saharan Africa (11). Moreover, there is a substantial funding gap in Africa’s HIV response; however, some countries like Botswana, Eswatini, Rwanda, Tanzania, and Zimbabwe are progressing toward the 95–95-95 targets. Despite this progress, a significant number of people and communities, including women, girls, and key populations, still lack access to HIV services due to gender inequalities, discrimination, violence, stigma, and harmful laws. Thus, the situation is worsened by the criminalization of HIV-positive individuals and high-risk groups, with many nations still restricting access to HIV-related health care for marginalized populations, which further exacerbates the burden of HIV (12).

To provide evidence-based HIV care and services in resource-constrained regions, comprehensive evidence is required. However, the available evidence is scarce and often not generalizable to the African region. Thus, comprehensive evidence using big data is quite important. Moreover, cluster-level factors are very important for appropriate public health interventions among the selected target groups. Therefore, this study aimed to estimate the prevalence of HIV and to identify its associated factors among women of reproductive age in Africa using the most recent Demographic and Health Survey (DHS) conducted in 2010 and later data.

## 2 Methods

### 2.1 Study setting, population, and data sources

**Figure 1:**
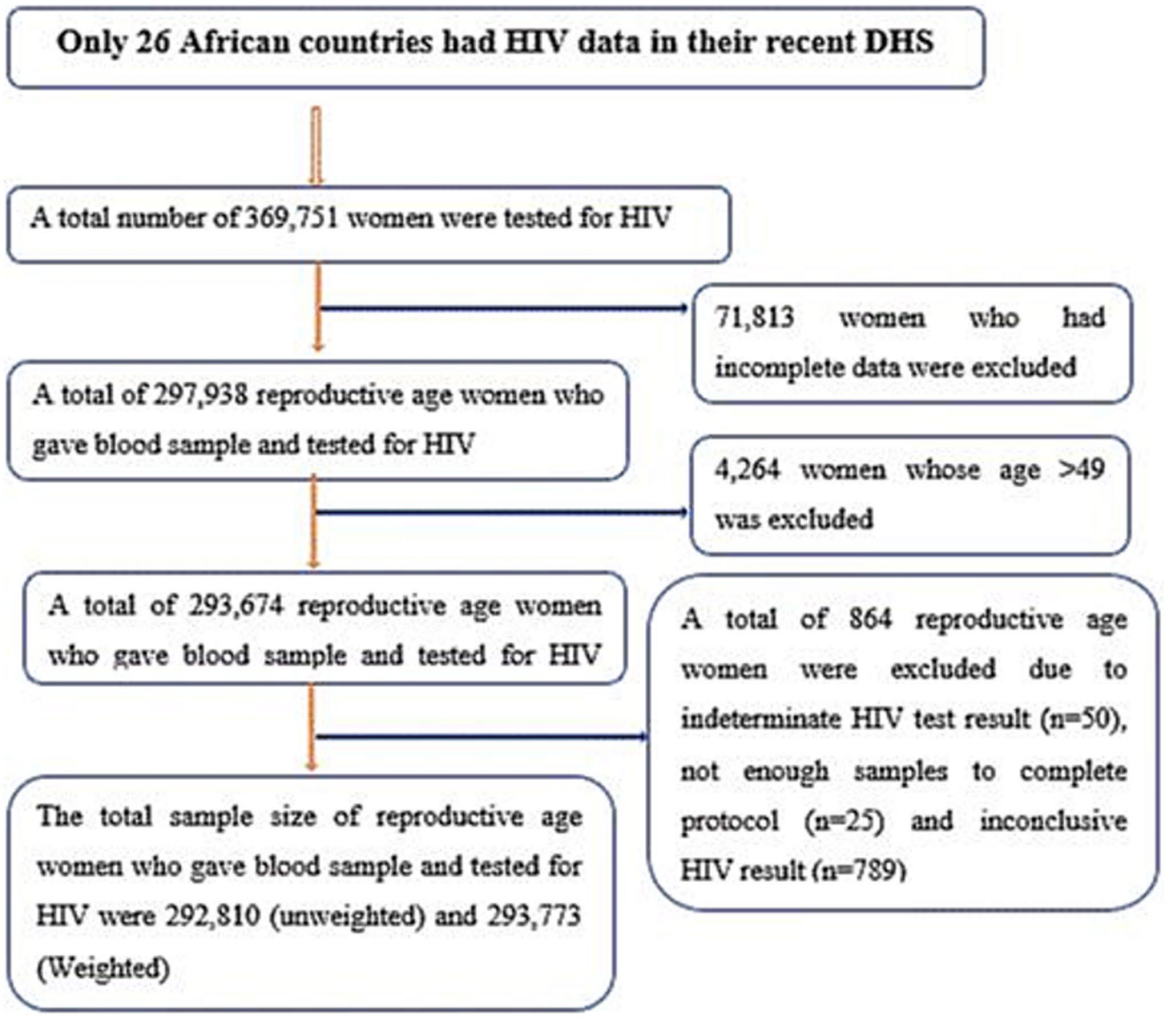
Schematic representation of the sampling procedure for this study.

This study is a secondary analysis of recent demographic and health survey (DHS) data from African countries. The DHS is conducted every 5 years. Among all African countries, only 26 countries had a record of routine HIV test results among reproductive-age women in their DHS dataset (Table 1, Figure 1). The DHS is primarily conducted in low- and middle-income countries and is a nationally representative survey. Data from each DHS were collected using a cross-sectional study design from 2010 to 2019, as shown in Table 1. Women living in Africa were considered the source population, while the study population for the current study consisted of women aged 15–49 years who lived in Africa and had complete HIV blood test results. The DHS dataset of 26 African countries was appended together to determine the burden of HIV and its drivers forces among reproductive-age women (15 to 49 years of age). For this study, we used an individual (women) record (IR) file and an HIV testing recode (AR) file. Finally, reproductive-age women who had complete HIV blood test results were included. In each DHS, a two-stage stratified sampling technique was used during participant selection. Enumeration areas (EAs) and households were selected in the first and second stages, respectively. The selection process for most DHSs involved unequal probabilities, requiring the use of weighting. Thus, we weighted our samples using the individual weight of women to increase the representativeness of the data. Weighting was performed by dividing the individual weight of women (v005) by 1 million. Data abstraction was carried out from 31 October 2023 to 30 November 2023, following the acquisition of the necessary ethical approval from the DHS. Consent from participants was waived for access to their previously collected data from the DHS.

**Table 1:** DHS survey years by country.

Country	Survey year	Country	Survey year
Angola	2015/16	Mali	2012/13
Burkina Faso	2010	Malawi	2015/16
Burundi	2016/17	Mozambique	2015
Democratic Republic of Congo	2013/14	Niger	2012
Cote d’Ivoire	2011/12	Namibia	2013
Cameroon	2018/19	Rwanda	2014/15
Ethiopia	2016	Sierra Leone	2019
Gabon	2019	Senegal	2017
Ghana	2014	Chad	2014/15
Gambia	2013	Togo	2013/14
Guinea	2018	South Africa	2016
Liberia	2013	Zambia	2018/19
Lesotho	2014	Zimbabwe	2015

### 2.2 Study variables

**Figure 2:**
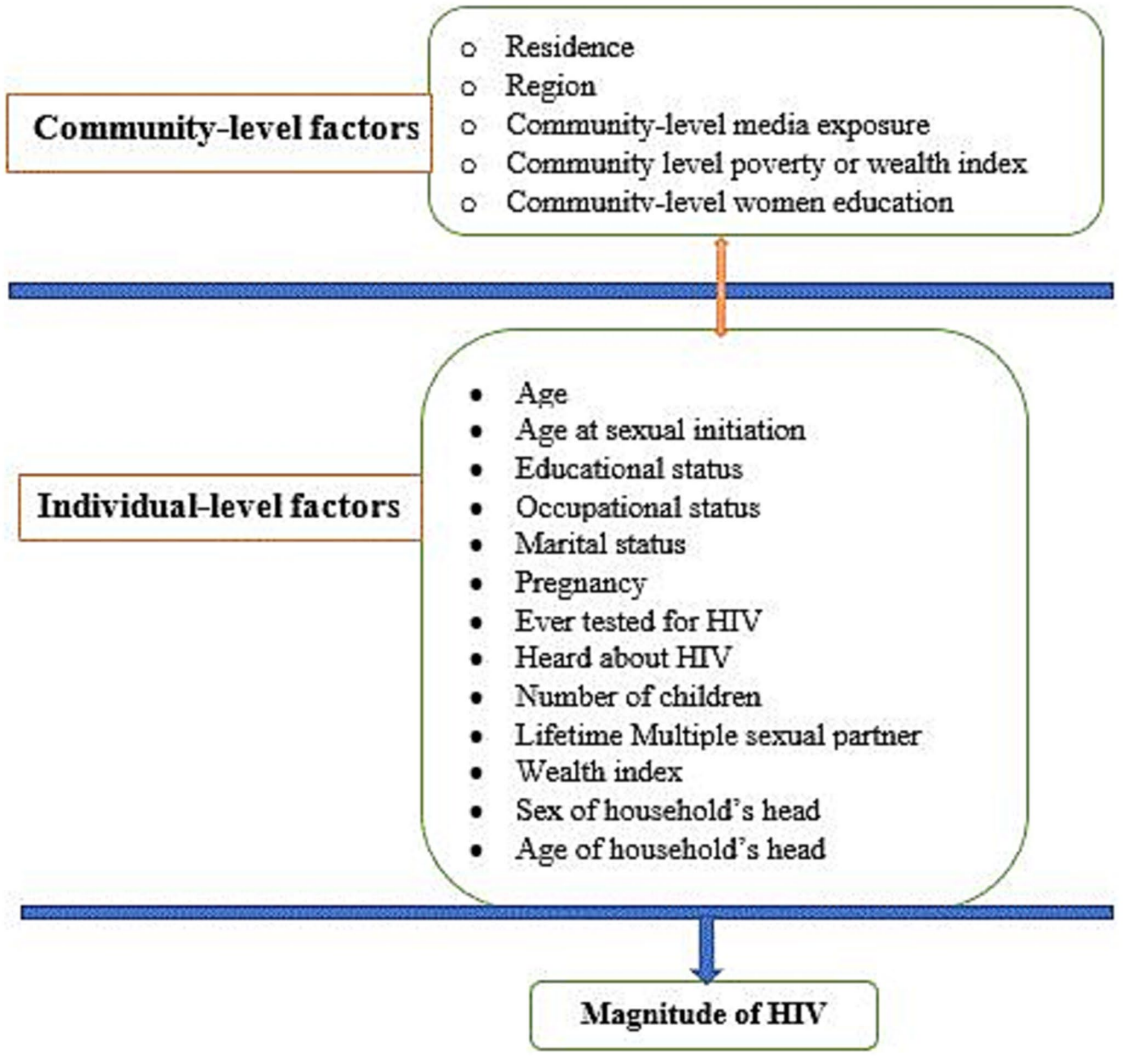
Conceptual framework showing factors associated with HIV among reproductive-age women in Africa.

The HIV test result was the outcome variable of this study, where it was classified as “Yes” when the HIV result of reproductive-age women was positive and coded as 1, otherwise “No” and coded as 0. Moreover, both individual and community-level data were considered as potential predictor variables of HIV prevalence among reproductive-age women in Africa. Individual-level (level I) variables included socio-demographic (age, marital status, educational status, occupational status, sex, and age of household head), HIV testing history, pregnancy status, age at first sexual intercourse, and economic characteristics (wealth index). Community-level (level II) variables included common characteristics of study subjects in an enumeration area, such as countries in the African region (East, West, Central, and South African regions), community-level media exposure (exposed versus unexposed), community-level women’s education (low versus high), community-level poverty or wealth index (low versus high), and residence (urban versus rural) (Figure 2).

### 2.3 Operational definition of the variables

- Community-level media exposure: It is defined as the exposure to media among women who were either listening to the radio, reading a newspaper or magazine, watching television, or using the Internet at least once a week (13).
- Community-level poverty or wealth index: It is defined as higher and lower poverty using the mean value of individual women’s wealth index.
- Community-level women’s education: It is defined based on the average proportion of educational level in the community as lower and higher educational attainment.

### 2.4 Data management and statistical analysis

The variables were extracted from IR and AR files, and STATA version 17.0 was used to clean, recode, and analyze the data. The individual (women’s) recode (IR files) from each of the 26 African countries was first merged with the HIV testing recode (AR files). Then, the merged IR and AR files from each of the 26 countries were pooled by appending the datasets together. The dataset was weighted using sample weights to compensate for the unequal probability of the strata selection process. Taking into account the hierarchical character of the data, a multilevel mixed-effects model was used to assess the fitness of the model for performing multilevel analysis. Thus, the measure of variation or random effect between clusters was assessed using the intra-cluster correlation coefficient (ICC), the median odds ratio (MOR), and the proportional change in variance (PCV) (14). The intraclass correlation coefficient (ICC), which shows the degree of heterogeneity in HIV prevalence between clusters, was calculated using the formula: ICC = $\frac{\delta 2}{\delta 2+\pi /3}$ ; ICC = $\frac{\delta 2}{\delta 2+3.29}$ , where δ2 indicates the estimated variance of the clusters. The MOR between two women with higher and lower propensity among two randomly chosen clusters can be compared, and it measures unexplained cluster heterogeneity of HIV distribution, which is calculated as $\mathrm{MOR}=\exp\left(\sqrt{2*\delta 2}\;*0.6745\right)\;\mathrm{or}\;\mathrm{MOR}=\exp\left(0.95*\delta \right)$ , where δ^2^ is the cluster-level variance. Moreover, the PCV measured the total variation in HIV distribution explained by individual- and community-level variables, which was calculated as PCV = $\frac{\delta 2\mathrm{null model}-\delta 2\;\mathrm{of each model}}{\;\delta 2\mathrm{null model}\;}$ , where δ2 of the null model was used as a reference.

A multilevel mixed-effects regression model was computed to find predictors of HIV among reproductive-age women in Africa. To detect the existence of a potential contextual effect, four models were fitted, the first being a null model (model I), the second being an adjustment for individual-level variables (model II), the third being an adjustment for community-level variables (model III), and the fourth being an adjustment for both individual and community-level variables (model IV). Deviation (−2Log likelihood), Akaike’s Information Criterion (AIC), and Bayesian Information Criterion (BIC) were used to compare the models because the models were nested. Finally, the fourth model (model IV) with the smallest information criterion value was chosen as the final best-fit model. In the final model, both community- and individual-level variables with a *p*-value ≤0.25 in the bi-variable analysis were included in the multivariable model. Finally, variables with an adjusted OR (AOR) with 95% CI and *p* < 0.05 were considered statistically significant predictors of HIV among reproductive-age women in Africa. Furthermore, multicollinearity was examined using the variance inflation factor (VIF) test and tolerance. All variables had a VIF < 10 and tolerance less than 1, indicating that multicollinearity did not exist.

### 2.5 Ethical considerations

Ethical approval was obtained from the Research and Ethics Review Committee of the Wollo University, College of Medicine and Health Sciences. The data from the Demographic and Health Survey were used for this study with permission obtained from the Measure DHS program at https://dhsprogram.com/ after registering and submitting a request with a brief statement of the objectives of the study. The data were used only for this registered research and cannot be shared with other researchers.

## 3 Results

### 3.1 Socio-demographic characteristics and HIV distribution

A total of 292,810 unweighted reproductive-age women were included in this study. Approximately 35, 398 (20.1%) of the women were aged between 35 and 39 years, which was the largest group. On average, the women reported an age at first sexual intercourse of 13.6 years, with a standard deviation of 7.17 years. The average age at first birth was 19.3 years, with a standard deviation of 3.88 years. Similarly, 45,014 (15.3%) women had no previous history of sexual exposure, of whom 1,705 (19.8%) women were found to be HIV positive. Approximately 46.4% of the women had a history of multiple sexual partners, and 5.6% of these women tested positive for HIV, compared to 3.5% of women without multiple partners. In terms of wealth index, 51,895(17.7%) and 54,208(18.5%) of women, respectively were poorest and poorer. Furthermore, 98,366 (31.5%) women had no formal education, of which 2.0% of them were HIV positive (Table 2).

**Table 2:** Background characteristics of study participants and HIV status by various background characteristics among reproductive-age women in Africa.

Variable	Category	Weighted frequency	HIV status	
			Negative no (%)	Positive no (%)
Women’s age	Mean ± SD	28.5 ± 9.45		
Women’s age at first delivery	Mean ± SD	19.3 ± 3.88		
Women’s age at first sexual intercourse	Mean ± SD	13.6 ± 7.17		
Head of household	Mean ± SD	44.6 ± 14.28		
Maternal age	15–19	61,994 (21.1)	59,322 (95.7)	2,672 (4.3)
	20–24	54,561 (18.6)	52,104 (95.5)	2,457 (4.5)
	25–29	51,706 (17.6)	49,536 (95.8)	2,170 (4.2)
	30–34	42,683 (14.5)	40,929 (95.9)	1754 (4.1)
	35–39	35,398 (20.1)	33,821 (95.6)	1,576 (4.4)
	40–44	26,365 (8.9)	25,207 (95.6)	1,158 (4.4)
	45–49	21,067 (7.2)	20,100 (95.4)	966 (4.6)
Age at first sexual intercourse	Did not have sex	45,014 (15.3)	43,309 (80.2)	1705 (19.8)
	<15 yrs	141 (0.05)	125 (88.7)	16 (11.3)
	>15	66,741 (22.7)	63,731 (95.5)	3,010 (4.5)
	Did not remember	181,876 (61.9)	173,854 (95.6)	8,022 (4.4)
Total number of children delivered	0	80,519 (27.4)	77,138 (95.8)	3,381 (4.2)
	1–3	115,781 (39.4)	109,822 (94.9)	5,959 (5.1)
	>3 (mean)	97,472 (33.2)	94,060 (96.5)	3,412 (3.5)
Maternal education	Illiterate	98,366 (31.5)	96,374 (98.0)	1991 (2.0)
	Primary	86,453 (29.4)	82,038 (94.9)	4,415 (5.1)
	Secondary	95,649 (32.6)	90,048 (94.1)	5,602 (5.9)
	Higher	13,305 (4.5)	12,560 (94.4)	745 (5.6)
Ever tested for HIV	No	138,700 (47.3)	134,994 (97.3)	3,706 (2.7)
	Yes	154,572 (52.7)	145,538 (94.2)	9,034 (5.8)
MSP	No	133,317 (53.6)	128,689 (96.5)	4,628 (3.5)
	Yes	115,277 (46.4)	108,870 (94.4)	6,407 (5.6)
Sex of head of household	Male	210,333 (71.6)	202,186 (96.1)	8,147 (3.9)
	Female	83,440 (28.4)	78,834 (94.5)	4,606 (5.5)
Currently pregnant	No	268,462 (91.4)	256,669 (95.6)	11,793 (4.4)
	Yes	25,311 (8.6)	24,351 (96.2)	960 (3.8)
Heard about HIV	No	15,691 (5.5)	15,252 (97.2)	439 (2.8)
	Yes	267,107 (94.5)	255,058 (95.5)	12,049 (4.5)
Marital status	Not formerly in a union	85,926 (29.2)	81,611 (95.0)	4,315 (5.0)
	In a union	207,847 (70.8)	199,409 (95.9)	8,438 (4.1)
Wealth index	Poorest	51,895 (17.7)	49,724 (95.8)	2,171 (4.2)
	Poorer	54,208 (18.5)	51,847 (95.6)	2,361 (4.4)
	Middle	56,640 (19.3)	54,203 (95.7)	2,437 (4.3)
	Richer	61,920 (21.0)	59,071 (95.4)	2,849 (4.6)
	Richest	69,110 (23.5)	66,174 (95.8)	2,936 (4.2)
Residence	Urban	118,633 (40.4)	112,991 (95.2)	5,642 (4.8)
	Rural	175,140 (59.6)	168,029 (95.9)	7,111 (4.1)
Media exposure	Exposed	92,308 (31.4)	88,794 (96.2)	3,514 (3.8)
	Not exposed	201,465 (68.6)	192,226 (95.4)	9,239 (4.6)
Occupation	Unemployed	128,564 (43.8)	122,047 (94.9)	6,517 (5.1)
	Employed	165,209 (56.2)	158,973 (96.2)	6,236 (3.8)
Ever heard of sexually transmitted infections (STIs)	No	13,462 (4.8)	13,101 (97.3)	362 (2.7)
	Yes	269,319 (95.2)	257,193 (95.5)	12,126 (4.5)

### 3.2 HIV among reproductive-age women (15–49 years) in Africa

The prevalence of HIV among reproductive-age women in Africa was 4.34% (95% CI: 4.2, 4.4%). The predominant frequency of HIV was reported in Lesotho (23.98%), followed by South Africa (19.12%) and Mozambique (14.67%). In contrast, less than 1% of HIV prevalence was reported in Niger (0.54%), Senegal (0.59%), and Burundi (0.79%) (Table 3). Among African sub-regions, the highest proportion of reproductive-age women tested for HIV was found in Western Africa (39.4%) and the lowest was in Southern Africa (7.4%). HIV was predominant in Southern African (18.5%) followed by Eastern African (6.1%) countries. The lowest level of HIV was observed in Western African countries (Table 3).

**Table 3:** HIV status by country of residence among reproductive-age women in Africa.

Country	Distribution of study participants					
	HIV positive		HIV negative		Total	
	Frequency	Percent	Frequency	Percent	Frequency	Percent
Angola	282	2.29	12,036	97.7	12,318	4.2
Burkina Faso	170	1.05	15,951	98.9	16,121	5.5
Burundi	136	0.79	17,085	99.2	17,221	5.9
Democratic Republic of Congo	200	1.11	17,802	98.9	18,002	6.1
Cote d’Ivoire	361	3.74	9,279	96.3	9,640	3.3
Cameroon	410	3.03	13,110	97.0	13,520	4.6
Ethiopia	234	1.49	15,446	98.5	15,680	5.3
Gabon	398	4.02	9,497	96.0	9,895	3.4
Ghana	156	1.71	8,941	98.3	9,097	3.1
Gambia	141	1.69	8,189	98.3	8,330	2.8
Guinea	131	1.46	8,847	98.5	8,978	3.1
Liberia	120	1.42	8,343	98.6	8,463	2.9
Lesotho	1,508	23.98	4,781	76.0	6,289	2.1
Mali	94	1.02	9,088	99.0	9,182	3.1
Malawi	1,340	9.55	12,688	90.4	14,028	4.8
Mozambique	1,013	14.67	5,890	85.3	6,903	2.3
Niger	48	0.54	8,917	99.5	8,965	3.1
Namibia	1,186	13.89	7,353	86.1	8,539	2.9
Rwanda	355	2.63	13,142	97.4	13,497	4.6
Sierra Leone	219	1.64	13,115	98.4	13,334	4.5
Senegal	86	0.59	14,411	99.4	14,497	4.9
Chad	189	1.62	11,442	98.4	11,631	4.0
Togo	191	2.05	9,127	98.0	9,318	3.2
South Africa	1,300	19.12	5,498	80.9	6,798	2.3
Zambia	1,536	11.29	12,064	88.7	13,600	4.6
Zimbabwe	949	9.56	8,978	90.4	9,927	3.4
African sub-region						
Eastern Africa	5,563	6.1	85,292	93.9	90,855	30.9
Central Africa	1,479	2.3	63,887	97.7	65,366	22.3
Southern Africa	3,995	18.5	17,633	81.5	21,628	7.4
Western Africa	1716	1.5	114,208	98.5	115,924	39.4
Total	12,753	4.34	281,020	95.7	293,773	100

### 3.3 Multilevel mixed-effects analysis of HIV among reproductive-age women in Africa

The random effects analysis showed that the ICC value in the null model (model I) was 0.49, indicating that 49% of the variability in HIV status was due to between-cluster/EA variability, while 51% was attributable to individual differences. Moreover, the full model accounted for 35% of the variation in HIV status among reproductive-age women in Africa. The variance between clusters (EA) in the null model (model I) was also 3.17, showing significant variability in HIV status. After accounting for individual and community-level factors, model IV revealed substantial variability in the odds of developing an HIV infection among women of reproductive age in Africa. This finding was further supported by the MOR results. Model fitness was evaluated using -2Log likelihood, Akaike’s Information Criterion (AIC), and Bayesian Information Criterion (BIC). Finally, model IV was chosen as the best-fit model as it had the lowest AIC (83249.11), the lowest BIC (83433.08), and the highest Log-likelihood (−41606.55) (Table 4).

**Table 4:** Multivariable multilevel mixed effect analysis results of individual- and community-level factors associated with the prevalence of HIV among reproductive-age women in Africa.

Variable	Category	Model II	Model III	Model IV
Maternal age	15–19	1		1
	20–24	0.95 (0.87–1.04)		1.01 (0.92–1.1)
	25–29	0.98 (0.88–1.1)		1.07 (0.96–1.19)
	30–34	1.04 (0.93–1.2)		1.1 (1.04–1.31)
	35–39	1.22 (1.09–1.38) **		1.4 (1.2–1.57) ***
	40–44	1.27 (1.12–1.44) ***		1.5 (1.3–1.7) ***
	45–49	1.47 (1.28–1.68) ***		1.75 (1.5–2.04) ***
Maternal education	Illiterate	1		1
	Primary	2.2 (2.1–2.38) ***		2.25 (2.1–2.4) ***
	Secondary	2.17 (1.99–2.37) ***		2.45 (2.2–2.7) ***
	Higher	1.98 (1.73–2.29) ***		2.49 (2.16–2.9) ***
Ever tested for HIV	No	1		1
	Yes	1.9 (1.8–2.1) ***		1.08 (1.0–1.16)
Multiple sexual partners	No	1		1
	Yes	1.27 (1.2–1.3) ***		1.3 (1.26–1.41) ***
Sex of head of household	Male	1		1
	Female	1.14 (1.08–1.2) ***		1.01 (0.95–1.05)
Currently pregnant	No	1		1
	Yes	0.9 (0.8–1.1)		0.97 (0.87–1.07)
Heard about HIV	No	1.32 (1.1–1.5) **		1.08 (0.9–1.25)
	Yes	1		1
Union status	Never in a union	1.21 (1.1–1.3) ***		0.9 (0.85–0.98) **
	In a union	1		1
Occupation	Unemployed	1.42 (1.35–15) ***		1.1 (1.04–1.17) **
	Employed	1		1
Wealth index	Lower		1	1
	Higher		1.15 (1.07–1.22) ***	1.14 (1.04–1.25) **
Residence	Urban		1	1
	Rural		0.77 (0.7–0.84) ***	0.8 (0.73–0.87) ***
Media exposure	Exposed		1	1
	Not exposed		1.02 (0.96–1.08)	1.03 (0.96–1.1)
Sub-region	East Africa		4.6 (4.2–5.0) ***	4.13 (3.76–4.5) ***
	Central Africa		1.4 (1.3–1.6) ***	1.18 (1.05–1.34) **
	South Africa		14.3 (13.1–15.6) ***	10.6 (9.5–11.8) ***
	West Africa		1	1
Women’s education	Lower		1	1
	Higher		1.12 (1.05–1.2) ***	1.17 (1.09–1.31) *
Random effects model				
ICC (%)	0.49	0.42	0.37	0.35
Variance (SE)	3.17 (1.0051)	2.34 (0.71682)	1.97 (0.77132)	1.78 (0.82684)
MOR (%)	Exp (3.0115)	Exp (2.223)	Exp (1.8715)	Exp (1.691)
PCV	Reference	26.2%	37.8%	43.8%
Model fitness				
-2Log likelihood	−56,819	−45253.36	−50005.61	−41606.55
AIC	113643.8	90534.72	100023.2	83249.11
BIC	113665.1	90680.92	100085.5	83433.08

*p < 0.05, **p < 0.01, ***p < 0.001; AIC, Akaike’s Information Criterion; BIC, Bayesian Information Criterion; ICC, Intra-cluster correlation coefficient; MOR, median odds ratio; PCV, proportional change in variance.

Model I is the null model, a baseline model without any explanatory variable.

Model II is adjusted for the individual level.

Model III is adjusted for community-level variables.

Model IV is the final model adjusted for all explanatory variables and country of survey.

In the fixed effects analysis of the final model (model IV), multiple factors from individual and community-level variables were statistically associated with HIV status. The likelihood of developing HIV infection was 2–2.49 times higher among women who had a higher educational status [AOR, 2.49, 95% CI (2.16, 2.9)] than among women with no education. Similarly, the odds of developing HIV infection among reproductive-age women whose age group ranged from 35to 39, 40 to 44, and 45 to 49 years were 1.4, 1.5, and 1.75 times higher, respectively, than among women aged 15–19 years. Moreover, reproductive-age women who were not in a union were 10% less likely to develop HIV infection compared to those who were in a union [AOR, 0.9, 95% CI (0.85, 0.98)]. The odds of developing HIV among women who had a lifetime history of multiple sexual partners were 1.3 times higher than those who did not have multiple sexual partners [AOR, 1.3, 95% CI (1.26, 1.41)]. Additionally, women who were unemployed had a 1.1 times higher likelihood of being HIV-positive than those who were employed [AOR, 1.1, 95% CI (1.04, 1.17)]. When considering community-level factors, reproductive-age women who had a higher wealth index and higher community-level education, respectively, were 1.14 times [AOR = 1.14; 95% CI (1.04, 1.25)] and 1.17 times [AOR, 1.17, 95%CI (1.09, 1.31)] more likely to be infected by HIV than their counterparts. Rural residence was associated with 80% lower odds of HIV infection compared to urban residence [AOR 0.8, 95% CI (0.73, 0.87)]. Furthermore, women who lived in Southern Africa, Eastern Africa, and Central Africa had a 10 times higher likelihood [AOR, 10.6, 95% CI (9.5, 11.8)], a four times higher likelihood [AOR, 4.13, 95% CI (3.76, 4.5)], and a 1.18 times higher likelihood [AOR, 1.18, 95% CI (1.05, 1.34)], respectively, of being HIV-positive than women who lived in Western African countries (Table 4).

## 4 Discussion

This study aimed to investigate the prevalence of HIV and its individual and community-level determinants among reproductive-age women in Africa based on DHS data collected from 2010 to 2019. The overall findings of the present study reveal that a significant regional variation in HIV among African countries is associated with both individual and community-level factors, which further indicates the complex nature of HIV transmission.

The overall prevalence of HIV among reproductive-age women in Africa was 4.34% (95% CI: 4.2, 4.4%), which was lower than the 6.5% HIV prevalence in SSA (15). The current point estimate suggests that the prevalence of HIV declined more rapidly in this target group compared to surveys conducted from 2003 to 2012 in SSA. This variation could be attributed to differences in the time periods, where different public health interventions implemented after 2012 may have contributed to a reduction in HIV transmission. Moreover, the findings of the current study showed a higher prevalence than the 0.85% prevalence of HIV reported in Ethiopia (9). The higher prevalence in the current study may be due to the inclusion of data from highly HIV-endemic areas, which likely contributed to the increased HIV prevalence in South African countries. Furthermore, the prevalence of HIV in this study was lower than the 10.3% HIV prevalence reported in Mozambique (16). This could be because the prevalence of HIV burden was higher in the Southern region of SSA where Mozambique is located (17). This finding also suggests the role of geographic locations in the transmission of HIV.

The predominant burden of HIV was reported in Lesotho (23.98%), which is consistent with the 27.9% HIV prevalence reported in a recent 2020 Lesotho Population-Based HIV Impact Assessment (LePHIA) among women. This finding implies that the burden remains alarming, with more than one-fourth of women from Lesotho infected with HIV. Similarly, the second predominant prevalence of HIV was found in South Africa (19.12%). This statistical figure suggests that nearly one in five South African women is living with HIV. Thus, the burden of HIV in this region is enormous and requires an integrated, multisectoral collaboration to alleviate HIV transmission. Furthermore, the lowest prevalence of HIV was reported in Niger (0.54%) and Senegal (0.59%), which is supported by previous findings that reported HIV prevalence ranging from 0 to 2% (18).

The current study confirmed that the predominant HIV burden in the Southern African region (18.5%) was followed by Eastern African countries (6.1%). The high burden of HIV in the Southern African region could be due to the socio-political environment, cultural variations, and risk behaviors that increase vulnerability to HIV acquisition and transmission. Furthermore, this rate suggests that the overall decline is not yet fast enough to achieve the UNDP’s 2025 95–95-95 targets, and may be an indication of the failure of targeted public health interventions in the region (19). Moreover, the lowest HIV prevalence was found in the Western African region (1.5%), which may be explained by the effectiveness of targeted HIV prevention interventions among key populations such as female sex workers (20).

Moreover, women who had a history of multiple sexual partners (more than two) had approximately a 1.3 times higher likelihood of HIV infection than their counterparts. This may be due to the fact that HIV infection is a sexually transmitted infection (STI), and the risk of transmission increases with the duration of sexual activity and the number of sexual partners. As a result, these behaviors encourage the spread of STIs, particularly HIV. Therefore, having several sexual partners in a close-knit social network increases the chance of contracting STIs, as it facilitates the virus’s rapid transmission (21). If multiple sexual partnerships are due to engagement in transactional sex for material or financial benefits, women may be less likely to insist on safer sex practices, thereby increasing the risk of HIV transmission. Similarly, the odds of HIV infection were lower among women who were never in a union than those women who were in a union or living together. This could be due to higher sexual exposure among women in a union, which is the key mode of HIV transmission.

Furthermore, women with higher educational status had an increased likelihood of HIV infection than their counterparts and this was statistically significant. This evidence disagreed with findings from South Africa (22), which may be due to the risk exposure difference of the women included in the study, along with the change in the study period. The statistical linkage may be due to the fact that more educated women are likely to be wealthier and more mobile, migrating from rural to urban areas where HIV is more endemic. Additionally, they may have more sexual partners or networks (23).

Similarly, unemployed women had a higher likelihood of acquiring HIV than their counterparts, which is supported by a previous study in SSA (24). This could be because unemployed women are more likely to be the poorest, and they may engage in high-risk behaviors including commercial sex work to alleviate their financial problems, which further increases their vulnerability to acquiring HIV infection. As a result, the odds of HIV infection were higher among women with a higher wealth index, which is supported by previous findings in Ethiopia (9) and SSA (24). Women with a higher wealth index are more likely to be educated and mobile, often living in urban areas where the endemicity of HIV is high.

The prevalence of HIV was lower among rural women than among urban dwellers, showing a disproportionately higher concentration of HIV/AIDS found in urban areas, in agreement with the findings of existing studies (9, 25, 26). This disproportionately lower prevalence in rural areas may be due to the expansion of injecting drug use, increased rates of drug-related activities, and levels of social interaction, all of which can significantly impact the patterns of women’s sexual interaction. Moreover, commercial sex activities (9, 25) and non-regular sexual relations are reported to be higher in urban areas than in rural ones. The population distribution, such as the male predominance in urban areas, may increase the extent of commercial sex activities. All of these conditions favor the transmission of HIV (25).

The rate of HIV among reproductive-age women varies considerably across Africa. The rate of infection is lower in West African countries than in other areas of SSA. However, the clear reasons for this change in epidemiological patterns of HIV/AIDS in Africa are not well understood. The most likely reasons for the increased likelihood of HIV infection exposure among South African women include limited access to sexually transmitted infection treatment opportunities, women engaging in heterosexual practices that increase the risk of recent infection, intergenerational sex, and the highest rates of pregnancy (27). Moreover, the effect of poverty may escalate the transmission of HIV in Eastern and Southern African countries, because HIV/AIDS can strike both the poor and the rich; however, the prevalence varies between these groups. In addition to this, the burden of financial problems in these regions may increase the vulnerability of women, because they may be exposed to the exchange of sex for money (28). Furthermore, the higher prevalence of HIV in South Africa is associated with ethnicity-based inequalities that accelerate the transmission of HIV in a certain marginalized population. The socio-political-economic system has been another driving force of HIV in this region (22, 29, 30). The implication of this study is to develop an appropriate evidence-based public health intervention of HIV among key populations in Africa. This study produced strong evidence using a large sample size, which may be significant for generalizability. However, the present study has certain limitations. For instance, due to the cross-sectional nature of the design employed by the DHS, causality could not be established. The time frame and the regional variation may be another limitation. Since the study uses data from 2010 to 2019, it may not reflect the current trend and status of HIV. The difference in sociocultural variations in Africa may not be equally applicable. Moreover, the trends of HIV over time in each country cannot be understood due to the lack of longitudinal data.

## 5 Conclusion

The prevalence of HIV among reproductive-age women was still high and was disproportionately higher in Southern African countries. Higher maternal education and wealth index, older maternal age, unemployment, sub-Saharan region, a history of multiple sexual partners, being in a union, and being an urban dweller were the predictors of HIV infection. Therefore, healthcare professionals and other concerned stakeholders should work on women’s empowerment, healthcare service expansion, and promoting their evidence-based public health intervention strategy for preventing the transmission of HIV among these key populations. Overall, a multisectoral response is warranted to meaningfully reduce the ongoing HIV burden faced by women across the African continent through women empowerment in education, economic opportunities, healthy relationships, and accessible prevention/treatment services.

## Data Availability

Publicly available datasets were analyzed in this study. This data can be found here: https://dhsprogram.com/.
